# Why can parents’ phubbing affect mobile phone addiction? Perspective of the I-PACE model

**DOI:** 10.3389/fpsyt.2024.1470306

**Published:** 2025-01-15

**Authors:** Bo Liu, Hemuqing Xu, Hua Wei, Yuancai Song

**Affiliations:** ^1^ Xinyang Normal University, Xinyang, China; ^2^ Department of Psychology, School of Education Science, Qingdao University, Qingdao, China

**Keywords:** parents’ phubbing, mobile phone addiction, maladaptive cognition, ego depletion, adolescent

## Abstract

**Objective:**

Based on the I-PACE model, this study investigated the longitudinal relationship between parents’ phubbing and mobile phone addiction, as well as the serial mediating effects of maladaptive cognition and ego depletion.

**Methods:**

A longitudinal study, with data collected at two time points over a six-month interval to test the proposed hypotheses. The independent variable was measured at Time 1 (T1), while the mediators and dependent variables were assessed at Time 2 (T2). A total of 591 middle school students from a city in central China was recruited to complete the parents’ phubbing scale, maladaptive cognition scale, ego depletion scale and mobile phone addiction scale.

**Results:**

(1) The correlation analysis showed a significant positive correlation between T1 parents’ phubbing, T2 maladaptive cognition, T2 ego depletion, and T2 mobile phone addiction. (2) Maladaptive cognition was found to mediate the relationship between parent’s phubbing and mobile phone addiction. Similarly, ego depletion also mediated the relationship between parent’s phubbing and mobile phone addiction. Additionally, both maladaptive cognition and ego depletion functioned as serial mediators between parent’s phubbing and mobile phone addiction.

**Conclusion:**

This study provides evidence that parental phubbing serves as a longitudinal predictor of mobile phone addiction, with maladaptive cognition and ego depletion acting as key mediators in this process.

## 1 Introduction

With the widespread use of smartphones and the development of digital technology, adolescent mobile phone addiction has emerged as an increasingly serious social issue, particularly pronounced in China. Mobile phone addiction can exert substantial negative effects on adolescents’ physical and mental health, social interaction, and academic performance (1–3). Therefore, it is of great practical significance to investigate the influencing factors and mechanisms of adolescent phone addiction. Parenting has an important impact on mobile phone addiction. Traditional parenting practices, such as harsh parenting, parental warmth, parental rejection, psychological control, and overprotection, have been found to be associated with mobile phone addiction (4–9). In addition, new parenting behaviors emerging in the digital age can also increase the risk of mobile phone addiction (10–15) Although some studies have noted the relationship between parents’ phubbing and mobile phone addiction, the mechanisms underlying this relationship remain underexplored. Therefore, This study aims to examine the mediating role of maladaptive cognition and ego depletion between parents’ phubbing behavior and mobile phone addiction based on the Interaction of Person-Affect-Cognition-Execution (I-PACE) model (16, 17).

According to the I-PACE model, addictive behaviors arise from the interaction between a person’ s predisposing variables, affective and cognitive responses to specific stimuli, and executive functions (16, 17). This model has been supported by numerous studies on addiction (15, 18, 19).The P-component of the model represents a person’s core characteristics likely involved in the addiction process as predisposing variables. These predisposing factors include not only individual attributes such as genetics, temperament, needs, and coping styles, but also family experiences. Therefore, parental phubbing, as part of an individual’s family experience, may also affect mobile phone addiction. The I-PACE model further posits that, beyond the direct effect of a person’s predisposing factors on addiction, affective and cognitive responses, alongside executive functions, serve as critical mediating mechanisms. Therefore, maladaptive cognition as a cognitive response to parent’s phubbing, and ego depletion as an indicator of impaired executive function, may mediate the relationship between parents’ phubbing and mobile phone addiction.

### 1.1 Parents’ phubbing and mobile phone addiction

Parents’ phubbing refers to the behavior of parents using their mobile phones during interactions with their children (10). The association between parent’s phubbing and adolescent mobile phone addiction can be understood through the lens of self-determination theory (20, 21). According to this theory, basic psychological needs are innate human needs, and people have a tendency to pursue the satisfaction of basic psychological needs. When the satisfaction of these needs is obstructed in one context, individuals may seek alternative means of fulfillment in other contexts. Mobile phone use, with its capacity to meet psychological needs, may become particularly addictive for individuals who are unable to achieve satisfaction in their real-life interactions.

Relationship needs are one of the basic psychological needs, and adolescents, who are particularly prone to feelings of loneliness, have a heightened desire to fulfill this need. Parents’ phubbing disrupts normal parent-child communication, leading adolescents to feel neglected and ignored, thus preventing their relationship needs from being effectively met (22–25). When adolescents fail to satisfy their relationship needs within the parent-child dynamic, they are likely to seek alternative forms of compensation. Unlike offline interactions, mobile social apps are specifically designed to provide psychological need satisfaction with an immediacy, consistency, and density that is difficult to find in other contexts (26). Immediacy refers to the ability of social apps to quickly and easily offer opportunities for relationship need fulfillment, accessible at nearly any time and place due to the mobility of smartphones. Ideally, these apps deliver minimal delay in the feedback or rewards resulting from social interactions. Consistency suggests that these apps reliably and predictably provide engagement and need satisfaction. Interactive features, such as likes and comments, ensure stable and continuous positive feedback. Density reflects the idea that mobile apps are designed to satisfy needs at a high frequency, far exceeding what can typically be achieved in everyday offline contexts. Through these apps, users can engage with large numbers of friends simultaneously, offering a level of social density and feedback that offline interactions cannot match. Given the aforementioned advantages of mobile social apps, adolescents who are unable to fulfill their relationship needs due to parental phubbing may turn to mobile phones for alternative satisfaction. Overreliance on this form of gratification can easily lead to addiction. Previous empirical studies have found positive associations between parents’ phubbing and mobile phone addiction (10–15, 27, 28).

Thereby, we propose H1: Parents’ phubbing is positively associated with mobile phone addiction.

### 1.2 Parents’ phubbing and mobile phone addiction: serial mediation of maladaptive cognition and ego depletion

To effectively address mobile phone addiction, it is essential not only to focus on the relationship between parents and mobile phone addiction but also to explore the underlying mechanisms. According to the I-PACE model and related studies, this study will examine the serial mediating roles of maladaptive cognition and ego depletion. The I-PACE model states that core personal characteristics (including family-related factors) affect executive function through emotional and cognitive responses, ultimately leading to addictive behavior (16, 17).Specifically to this study, we hypothesized that parents’ phubbing (personal core characteristics) will have a relationship with mobile phone addiction through the serial mediation of maladaptive cognition (cognitive response) and ego depletion (executive function).

First, there may be a positive correlation between parents’ phubbing and maladaptive cognition. Maladaptive cognition refers to a distorted belief system in which individuals perceive the virtual world as superior to the real world (29).Parents are the main source of social support for adolescents in the real world, and parent’s phubbing can lead to feelings of neglect in adolescents (30), thereby fostering a negative perception of the real world. Such negative feedback from parents may contribute to the formation of negative self-cognition, making adolescents feel unnoticed and undervalued in their everyday lives. In contrast, the virtual world offers immediate and positive reinforcement through likes, comments, and replies, making it easier for adolescents to feel a sense of importance and value. Consequently, they may develop the distorted belief that the virtual world is superior to the real one. Recent studies corroborate this perspective, researchers finding that higher levels of parent’s phubbing are associated with increased maladaptive cognition (31).

Second, maladaptive cognition is closely related to ego depletion. According to the strength model of self-control, many activities require self-control, such as thought suppression, emotional regulation and resisting temptation. These activities deplete an individual’s limited self-control resources, leading to a state of diminished self-control capacity, known as ego depletion (32, 33). Adolescents with high levels of maladaptive cognition often view the Internet as a more comfortable and appealing space compared to real life. The Internet’s allure motivates them to spend considerable time in the virtual world. However, Chinese middle school students typically face heavy academic tasks and strict discipline, leaving them limited time for online activities. Therefore, adolescents with high maladaptive cognition must frequently resist the temptation to use the Internet. Many studies have found that resisting temptation causes ego depletion (34, 35). For instance, research has found that individuals who resist the temptation to eat chocolate experience more ego depletion than those who give in to the temptation (34). Similarly, adolescents with high maladaptive cognition in this study are likely to experience ego depletion as they constantly resist the temptation of the Internet. The relationship between maladaptive cognition and ego depletion can also be explained from the perspective of the Self-Regulatory Executive Function (S-REF) model (36–38). According to this model, metacognitions about certain activities can affect extended thinking (including intrusive thinking), thereby impairing executive function. Maladaptive cognition, which reflects a distorted positive perception of Internet use, can be considered a form of positive metacognition regarding the Internet. Such metacognition may lead individuals to repeatedly imagine scenarios and experiences related to Internet use, generating intrusive thinking. These intrusive thinking disrupt ongoing activities, often requiring suppression. This suppression of thoughts further depletes self-control resources, contributing to ego depletion (33, 39). Previous empirical studies have also found a positive correlation between intrusive thinking and self-depletion (40).Therefore, Maladaptive cognition may also lead to ego depletion by increasing intrusive thinking.

Finally, ego depletion is closely related to mobile phone addiction. When in a state of ego depletion, individuals tend to disregard the future benefits of their actions, overlook the importance of long-term gains, and focus more on immediate rewards, making them more susceptible to addictive behaviors (41). Compared to real-life activities such as studying or exercising, mobile phone use provides adolescents with more immediate gratification. Therefore, adolescents experiencing ego depletion are likely to over-rely on their mobile phones for instant gratification, gradually developing habitual and uncontrollable mobile phone use through repeated negative reinforcement. This uncontrolled mobile phone use can reduce their engagement in academic activities and offline social interactions, ultimately damaging their academic performance and social relationships. In short, ego depletion leads to a loss of control over mobile phone use and impairs social functioning, both of which are core features of mobile phone addiction. Previous empirical studies have also found a positive link between ego depletion and both social networking addiction and Internet addiction, providing indirect support for the relationship between ego depletion and mobile phone addiction (42, 43).

Thereby, we propose H2: Maladaptive cognition and ego depletion would serially mediate the relationship between parents’ phubbing and mobile phone addiction.

## 2 Materials and methods

### 2.1 Participants and procedures

We conducted a two-wave study with 6-month intervals among Chinese adolescents between November 2022 and May 2023. During the first wave, data on the independent variables were collected, and in the second wave, the mediating and dependent variables were assessed. The initial sample consisted of 657 adolescents, including 269 females and 388 males. Six months later, at Time 2, attrition due to absenteeism or refusal to participate resulted in a decrease in participants from 657 to 607. Additionally, 16 students were excluded from the analysis due to providing uniform responses. Thus, the final sample included 591 middle school students, comprising 251 females (42.47%) and 340 males (57.53%), with ages ranging from 11 to 16 years (*M* = 13.82, *SD* = 0.79).

Based on the purpose of this study and sample availability, we have identified middle school students as the survey targets. Firstly, we contacted the school and described our research to them, obtaining their approval. After the school agreed to participate, we obtained the consent of the students, their parents/primary caregivers. Then, well-trained psychology graduate students and researchers distributed and collected questionnaires. The questionnaire is completed in the classroom using a pen and paper test. Before officially filling out the questionnaire, the researchers emphasized the rights of the participants, the anonymity and confidentiality of the survey. Finally, the researchers collected the questionnaire and expressed gratitude to the participants.

### 2.2 Measures

#### 2.2.1 Parents’ phubbing

We used the Chinese version of the Parent Phubbing Scale (44), which includes 9 items. Participants rated these items on a 5-point Likert scale ranging from 1 = Never to 5 = Always. The reliability and validity of this scale have been supported among Chinese students (27, 45). Cronbach’s alpha in this sample was 0.82 at T1.

#### 2.2.2 Maladaptive cognition

Maladaptive cognition was measured by a maladaptive cognition scale in Chinese, which includes 7 items (46). Participants evaluated the items on a 5-point Likert scale ranging from 1 = Never to 5 = always. The reliability and validity of this scale have been supported among Chinese students (46, 47). Cronbach’s alpha in this sample was 0.93 at T2.

#### 2.2.3 Ego depletion

We used the Chinese version of the ego depletion scale, which includes 5 items (48). Participants responded to these items using a 4-point Likert scale (from 1 = never to 4 = always).The reliability and validity of this scale have been supported among Chinese students (42, 43, 49, 50). Cronbach’s alpha in this sample was 0.88 at T2.

#### 2.2.4 Mobile phone addiction

We used the Chinese version of the mobile phone addiction scale, which includes 11 items (51). Participants were asked to assess the agreement of each item using a 6-point Likert scale from 1 (incompletely agreed) to 6 (completely agreed). The reliability and validity of this scale have been supported among Chinese students (15, 44, 52). Cronbach’s alpha in this sample was 0.78 at T2.

### 2.3 Data analysis

Missing data were addressed using the full information maximum likelihood estimator. SPSS 21.0 was utilized to perform descriptive statistics (i.e., mean and standard deviation of research variables) and person’ s correlation analysis between the variables of interest. All variables were standardized prior to analysis. Subsequently, the SPSS macro PROCESS 3.0 (model 6) was employed to assess the proposed serial mediation model (53). Mediation effect analyses were analyzed using the bootstrapping method, with 95% bias corrected confidence intervals and 5000 bootstrapped samples. Refer to recent research to determine the range of effect sizes, r = 0.1, r = 0.2, and r = 0.3 correspond to small, medium, and large effect sizes, respectively (54).

## 3 Results

### 3.1 Preliminary analyses

Pearson’s correlation analysis was used to examine the strength of the relationship between the predictor, mediator, and outcome variables. The results were shown in **Table 1**. There was a significant positive correlation between all variables. According to the new standard (54), the correlations between parents’ phubbing and maladaptive cognition(r = 0.22), parents’ phubbing and ego depletion(r = 0.27), parents’ phubbing and mobile phone addiction(r = 0.23) indicate medium effect sizes; the correlation between maladaptive cognition and ego depletion(r = 0.39), maladaptive cognition and mobile phone addiction (r = 0.34),ego depletion and mobile phone addiction(r =0.41) indicate large effects”.

**Table 1:** Correlation coefficients, means, and standard deviations of variables.

	*M*	*SD*	1	2	3	4
1. T1 Parents’ Phubbing	21.83	6.54	—			
2.T2 Maladaptive Cognition	10.03	4.99	0.22^**^	—		
3. T2 ego depletion	10.27	3.71	0.27^**^	0.39^**^	—	
4. T2 Mobile Phone Addiction	28.6	11.31	0.23^**^	0.34^**^	0.41^**^	—

*N*= 591. ** *p* < 0.01.

### 3.2 Mediating effect analyses

**Figure 1:**
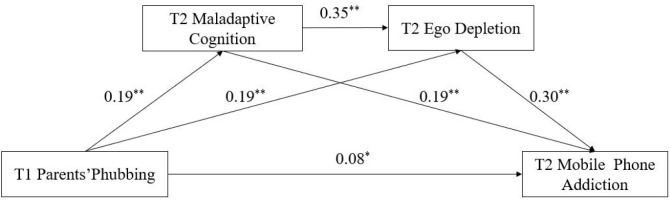
The serial mediating effects of parents’ phubbing and mobile phone addiction. * *p* < 0.05, ** *p* < 0.01.

To test the mediating hypothesis, we ran a PROCESS 3.0 (model 6) analysis to test the sequential mediating model. As shown in **Table 2** and **Figure 1**, after controlling for age and gender, T1 parents’ phubbing was positively associated with T2 maladaptive cognition (*β* = 0.19, *p* < 0.01, Equation 1). T1 parents’ phubbing (*β* = 0.19, *p* < 0.01, Equation 2) and T2 maladaptive cognition (*β* = 0.35, *p* < 0.01, Equation 2) were positively associated with T2 ego depletion. When T2 maladaptive cognition and T2 ego depletion were added as the mediators, T1 parents’ phubbing (*β* = 0.08, *p* < 0.05, Equation 3), T2 maladaptive cognition (*β* = 0.19, *p* < 0.01, Equation 3) and T2 ego depletion (*β* = 0.30, *p* < 0.01, Equation 3) were positively associated with T2 mobile phone addiction. The non-parameter bootstrapping simulation was used to test the indirect effects. **Table 3** shows the three indirect paths that were significant. These results indicated that T2 maladaptive cognition and T2 ego depletion were mediators in the relationship between T1 parents’ phubbing and T2 mobile phone addiction.

**Table 2:** The Mediation Effect Model.

	Equation 1 (criterion = Maladaptive Cognition T2)			Equation 2 (criterion = ego depletion T2)			Equation 3 (criterion = Mobile Phone Addiction T2)		
	*β*	*SE*	*t*	*β*	*SE*	*t*	*β*	*SE*	*t*
Gender	0.05	0.08	0.6	0.10	0.08	1.24	0.14	0.08	1.78
Age	0.16	0.05	3.04**	0.06	0.05	1.26	0.12	0.05	2.57*
T1 Parents’ Phubbing	0.19	0.04	4.67**	0.19	0.04	4.75**	0.08	0.04	2.11*
T2Maladaptive Cognition				0.35	0.04	8.85**	0.19	0.04	4.64**
T2 ego depletion							0.30	0.04	7.20**
T2 Mobile Phone Addiction									
*R*2	0.06			0.19			0.23		
*F*	11.98**			34.37**			33.26^**^		

* *p <*0.05, ** *p* < 0.01.

**Table 3:** Indirect Effects.

	Effect	95% Boot *CI*
T1Parents’Phubbing—T2Maladaptive Cognition—T2Mobile Phone Addiction	0.04	[0.02, 0.06]
T1Parents’Phubbing—T2Ego Depletion—T2Mobile Phone Addiction	0.06	[0.03, 0.09]
T1Parents’Phubbing—T2Maladaptive Cognition—T2Ego Depletion — T2Mobile Phone Addiction	0.02	[0.01, 0.03]

## 4 Discussion

In this study, we investigated the relationship between parents’ phubbing and mobile phone addiction, exploring its underlying mechanisms based on the I-PACE mode. Our findings indicate a positive association between parental phubbing and mobile phone addiction, with maladaptive cognition and ego depletion serving as serial mediators in this relationship. Consequently, both hypotheses (H1 and H2) were supported.

### 4.1 Parents’ phubbing and mobile phone addiction

First, the present study found a positive link between parents’ phubbing and mobile phone addiction, which is consistent with the previous studies (10–15, 27, 28). Parenting style has an important impact on mobile phone addiction. Early studies predominantly focused on traditional parenting styles, such as harsh parenting and psychological control, and their effects on mobile phone addiction (9, 55). In recent years, scholars have begun to explore the role of parents’ phubbing—a new parenting style emerging in the digital age. Unlike harsh parenting and psychological control, parental phubbing does not involve physical or verbal violence, nor does it typically entail negative emotions such as anger during parent-child interactions. As a result, its detrimental effects may be easily overlooked by parents. However, the results of this study suggest that for parents’ phubbing, despite appearing harmless, may have serious consequences, including mobile phone addiction.

### 4.2 Serial mediation of maladaptive cognition and ego depletion

Based on the I-PACE model, this study found the serial mediating effects of maladaptive cognition and ego depletion in the relationship between parents’ phubbing and mobile phone addiction. The results of this study explain how parents’ phubbing affects mobile phone addiction through cognitive (anxiety) and executive function (ego depletion) mechanisms, thereby supporting and expanding the I-PACE model. While previous studies based on this model have emphasized the role of emotional and cognitive characteristics (15) or focused on the effects of executive function (56); few have explored the serial mediating effects of both cognitive characteristics and executive function. Compared with previous studies, the results of this study provide a more comprehensive representation of the I-PACE model, demonstrating how individual family characteristics (i.e., parental phubbing) affect addictive behavior through cognitive traits (maladaptive cognition) and executive function (ego depletion). Future research should further investigate whether parental phubbing has a relationship with mobile phone addiction through emotional characteristics and executive functions.

Previous studies have found that parents’ phubbing has a relationship with mobile phone addiction through the mediating effects of emotions (such as depression and loneliness) (11, 13), parent-child relationship (parent-child affinity) (27) and peer relationship (deviant peer interaction) (12). However, there has been limited attention to the roles of cognitive characteristics (maladaptive cognition) and executive function (ego depletion) in this context. While emotional regulation and the fulfillment of relational needs are significant factors in the process of adolescent mobile phone addiction, the impact of cognitive distortions and declines in executive function should not be overlooked. The results of this study enrich our understanding of how parents’ phubbing affects mobile phone addiction, suggesting that we should pay attention to cognitive reconstruction and executive function training in adolescents as part of intervention strategies for mobile phone addiction.

It is worth noting that although existing research has examined many individual mediating variables, there is a lack of comprehensive research, and future research needs to strengthen research in this field.

### 4.3 Limitations and implications

The current study has three limitations. First, the data collection relied on self-reported method, which may introduce self-response bias. To mitigate this issue, we implemented several strategies, such as ensuring the confidentiality of responses, emphasizing that there were no right or wrong answers to the questions, utilizing varied questionnaire formats, and guaranteeing anonymity. Future research should consider employing alternative methods, such as video coding of parental behavior during parent-child interactions. Second, the current research was conducted exclusively with a sample of students from mainland China, which may limit the generalizability of the findings. Therefore, future studies could benefit from cross-cultural comparisons to enhance the robustness of the results. Third, while the samples in this study were drawn from China, the mechanism through which parent’s phubbing has a relationship with mobile phone addiction was not examined from the perspective of traditional Chinese culture. Future research should continue to explore this relationship within the framework of traditional Chinese cultural.

Despite these limitations, the present study advances previous studies by revealing the serial mediation mechanisms underlying the link between parents’ phubbing and mobile phone addiction. Specifically, the present study is the first attempt to reveal the serial mediation effects of maladaptive cognition and ego depletion, providing insight into how and why parents’ phubbing leads to mobile phone addiction.

In addition, this study offers several practical implications. First, researchers should educate parents about the harm of parents’ phubbing to adolescents and encourage them to reduce this behavior. While many parents recognize the harm of harsh parenting, they may overlook the negative impact of non-violent practices like phubbing. It is crucial to help parents understand that, although phubbing may appear peaceful and does not immediately trigger intense parent-child conflicts or negative emotions, it can undermine the parent-child relationship and adolescents’ mental health over time. Second, parents can increase their psychological energy by practicing mindful parenting, which helps them remain attentive to their children during interactions. Recent studies have found that parenting burnout and work-family conflict can increase parents’ phubbing (57, 58). Concentrating on child-rearing can be challenging, especially when parents face stress and disappointment, leading them to turn to mobile phones for emotional relief. Mindful parenting can alleviate home and work pressures, enabling parents to resist the temptation of using phones during parent-child interactions (59, 60). Third, schools can organize various offline activities to help teenagers meet their psychological needs in real life and reduce maladaptive cognition. Adolescents often develop maladaptive cognition when they find offline life unfulfilling and turn to the internet for psychological satisfaction. Schools could arrange collective music, sports, and other activities aligned with adolescents’ developmental needs to enhance their sense of autonomy, relatedness, and competence, thereby countering maladaptive cognition. Finally, schools can make learning more engaging by improving teaching methods and reducing adolescents’ ego depletion during the learning process. Given the heavy academic workload on Chinese teenagers, they must focus for extended periods, which can easily lead to ego depletion. However, research shows that enjoyable tasks can reduce ego depletion (61). By incorporating gamification and other interactive teaching methods, teachers can make learning more enjoyable and help students conserve their self-control resources.

## Data Availability

The raw data supporting the conclusions of this article will be made available by the authors, without undue reservation.
